# Phenotyping analysis of maize stem using micro-computed tomography at the elongation and tasseling stages

**DOI:** 10.1186/s13007-019-0549-y

**Published:** 2020-01-04

**Authors:** Ying Zhang, Liming Ma, Jinglu Wang, Xiaodong Wang, Xinyu Guo, Jianjun Du

**Affiliations:** 1 Beijing Key Lab of Digital Plant, Beijing Research Center for Information Technology in Agriculture, Beijing Academy of Agriculture and Forestry Sciences, No. 11 Shuguang Huayuan Middle Road, Haidian District, Beijing, 100097 People’s Republic of China; 2 College of Information and Electrical Engineering, China Agricultural University, 17 Qinghua Donglu, Haidian District, Beijing, 100083 People’s Republic of China

**Keywords:** Maize stem, CT scanning, μPhenotyping, Vascular bundle, Level set, Function zone

## Abstract

**Background:**

Micro-computed tomography (μCT) bring a new opportunity to accurately quantify micro phenotypic traits of maize stem, also provide comparable benchmark to evaluate its dynamic development at the different growth stages. The progressive accumulation of stem biomass brings manifest structure changes of maize stem and vascular bundles, which are closely related with maize varietal characteristics and growth stages. Thus, micro-phenotyping (μPhenotyping) of maize stems is not only valuable to evaluate bio-mechanics and water-transport performance of maize, but also yield growth-based traits for quantitative traits loci (QTL) and functional genes location in molecular breeding.

**Result:**

In this study, maize stems of 20 maize cultivars and two growth stages were imaged using μCT scanning technology. According to the observable differences of maize stems from the elongation and tasseling stages, function zones of maize stem were firstly defined to describe the substance accumulation of maize stems. And then a set of image-based μPhenotyping pipelines were implemented to quantify maize stem and vascular bundles at the two stages. The coefficient of determination (R^2^) of counting vascular bundles was higher than 0.95. Based on the uniform contour representation, intensity-related, geometry-related and distribution-related traits of vascular bundles were respectively evaluated in function zones and structure layers. And growth-related traits of the slice, epidermis, periphery and inner zones were also used to describe the dynamic growth of maize stem. Statistical analysis demonstrated the presented method was suitable to the phenotyping analysis of maize stem for multiple growth stages.

**Conclusions:**

The novel descriptors of function zones provide effective phenotypic references to quantify the differences between growth stages; and the detection and identification of vascular bundles based on function zones are more robust to determine the adaptive image analysis pipeline. Developing robust and effective image-based phenotyping method to assess the traits of stem and vascular bundles, is highly relevant for understanding the relationship between maize phenomics and genomics.

## Background

With the advent of the first digital images in the 1960s [1], cell biologists began to use computer programs to manipulate their images in order to “better see what needs to be seen” [2]. The complexity and diversity in microscopic image data, however, poses challenges for developing suitable data analysis workflows. Micro-phenotypic screening based on machine learning has been a hot topic in cell biology, plant physiology and agricultural breeding in recent years. Machine learning aims to provide a general solution to this problem by learning processing rules from examples rather than relying on manual adjustments of parameters or pre-defined processing steps [3–6]. The machine learning methods used in microscopic image analysis mainly include neural networks [7], adaptive lifting [8], random forests [9] and support vector machines [10], formed with specialized image-analysis software such as Wndchrm [11], CellProfiler [12], CellCognition [13], EBImage [14], PhenoRipper [15], or ImageJ/Fiji [16, 17]. Most image analysis algorithms, however, have been developed for specific biological assays. For the specific crop organ and cell phenotypic characteristics of the huge differences, there generally need to be specially developed in order to truly meet the research needs [18].

Vascular system is an important aspect of stalk structure and responsible for both the delivery of resources (water, essential mineral nutrients, sugars and amino acids) to the various plant organs, and the provision of mechanical support. In addition, the vascular system serves as an effective long-distance communication system, with the phloem and xylem serving to input information relating to abiotic and biotic conditions above and below ground, respectively [19]. Better understanding the variations in vascular bundles within stems in different growth stages and different varieties will provide useful information for genetic improvement of yield potential. Over the past decade, considerable progress has been made in terms of our understanding of anatomical structure and function of maize vascular system. With the development of microscopic imaging technology and computer vision technology, the phenotypic characteristics and distribution pattern of vascular bundles have been extended from qualitative description to quantitative analysis [20]. Zhang et al. [21] developed an automated image analysis method for stained maize stem cross sections to quantify the lignification of stem tissues. Legland et al. [22] and Heckwolf et al. [23] created an image analysis tool that could operate on images of hand-cut stem transections to measure stem anatomical features and vascular bundles traits without the rind. Legland et al. [17] developed an image analysis procedure for histological quantification of maize stem sections from FASGA-stained images, such as the size and average coloration of lignified fraction and non-lignified fraction could be quantified. Those tools have significantly improved the measurement efficiency of stem morphological characteristics analysis, but the more sophisticated vascular bundle anatomical traits corresponding to the stem section and the detection accuracy remained a challenge. Du et al. [24] firstly introduced micro-computed tomography (CT) technology for stem imaging and developed the VesselParser 1.0 algorithm, which made it possible to automatically and accurately analyze phenotypic traits of vascular bundles within entire maize stalk cross-sections. Although several tools have become available as mentioned above, because of very variable tissue morphology and image resolution for different maize varieties in different growth stages and environments, more robust and adaptive image processing methods are urgently needed that are suitable for mass samples of different periods, varieties and environments.

In this study, we presented a set of image-based μPhenotyping pipelines to detect the phenotypic traits of basal stem internodes and vascular bundles at both the elongation and tasseling stages. Combined with μCT scanning technology, new micro-phenotypic parameters, such as intensity-related, geometry-related and distribution-related traits of vascular bundles were extracted and used to quantify dozens of stem samples at the different growth stages.

## Methods

### Material

20 maize varieties (Jingke968, Jingke665, Zhongdan909, Jingdan28, Zhengdan958, Xianyu335, Denghai605, Nongda108, Dika517, SK567, Xianyu696, Jundan28, Jingkenuo2000, Jingkenuo2010, Jingke9681, Jingdan58, Jingke9685, Jingkeqingzhu301, Jingketian183, and Jingnongke728) were grown in experimental field of Beijing Academy of Agriculture and Foresting Sciences (BAAFS) in Beijing, China, 2017. Each variety was planted in two-row plot, with 10 plants per row, and the inter-row spacing was 60 cm. The stem third-internodes of each variety with three replicates were collected at elongation and tasseling stages, respectively. Depending on the size of sample compartment, the third internode of maize stems were firstly cut into small segments with 1 to 1.5 cm by a motor electric cutting machine (Bosch stone cutting machine GDM13-34). The sample segments were then soaked in FAA solution (90:5:5 v/v/v, 70% ethanol:100% formaldehyde:100% acetic acid) immediately. After the FAA fixation, samples were performed the sequential ethanol gradient dehydration in batch (i.e., 70%, 95% and 100%) and set the processing time of each ethanol gradient as 30 min. Next, samples were transferred to tertiary butyl alcohol and soaked for 24 h, and then were frozen at − 80 °C for 24 h. Finally, frozen samples were placed in the freeze-dryer (LGJ-10E, China) and freeze-dried for 3 h in batch.

Dried stem sample was inserted in wax (3 M Unitek, Monrovia, CA 91016 USA), stacking together to the sample table to maintain it and avoid movement during scanning time. Then the sample was scanned by Skyscan 1172 (SkyScan 1172, Bruker Micro-CT), and the unified scanning parameters were set as: X-ray source energy of 40 kV, a current of 250 µA, the imaging pixel sizes with 13.55 µm, 2 K scanning mode (2000 × 2000 pixels), and no filter [25]. The distance between Object and Source was 259.850 mm, and the distance between Camera and Source was 345.591 mm. The scan time of each sample was 20 min. The sample was rotated over 180° with 0.4° rotation step during the image acquisition. At each angular position a shadow image or transmission image was acquired. The cone beam acquisition saved all these projection images as 16-bit TIFF files. The exposure time was 620 ms. A total of 1000 2D projection images was generated.

According to X-ray linear attenuation coefficient of various materials, the Hounsfield (HU) values of air and water are respectively − 1000 and 0. For different maize varieties and growth stages, we observed that HU values of maize stem distributed in a wide range from 0 to 7000. To provide a quantification and evaluation standard for the properties of maize stems, we defined a wider value range which could cover whole HU ranges of plant materials, i.e. [− 1000, 9240], to transform raw data (16-bit TIFF) into an 8-bit grey-level image with a value range [0, 255].

### Function zones identification of maize stem

**Fig. 1:**
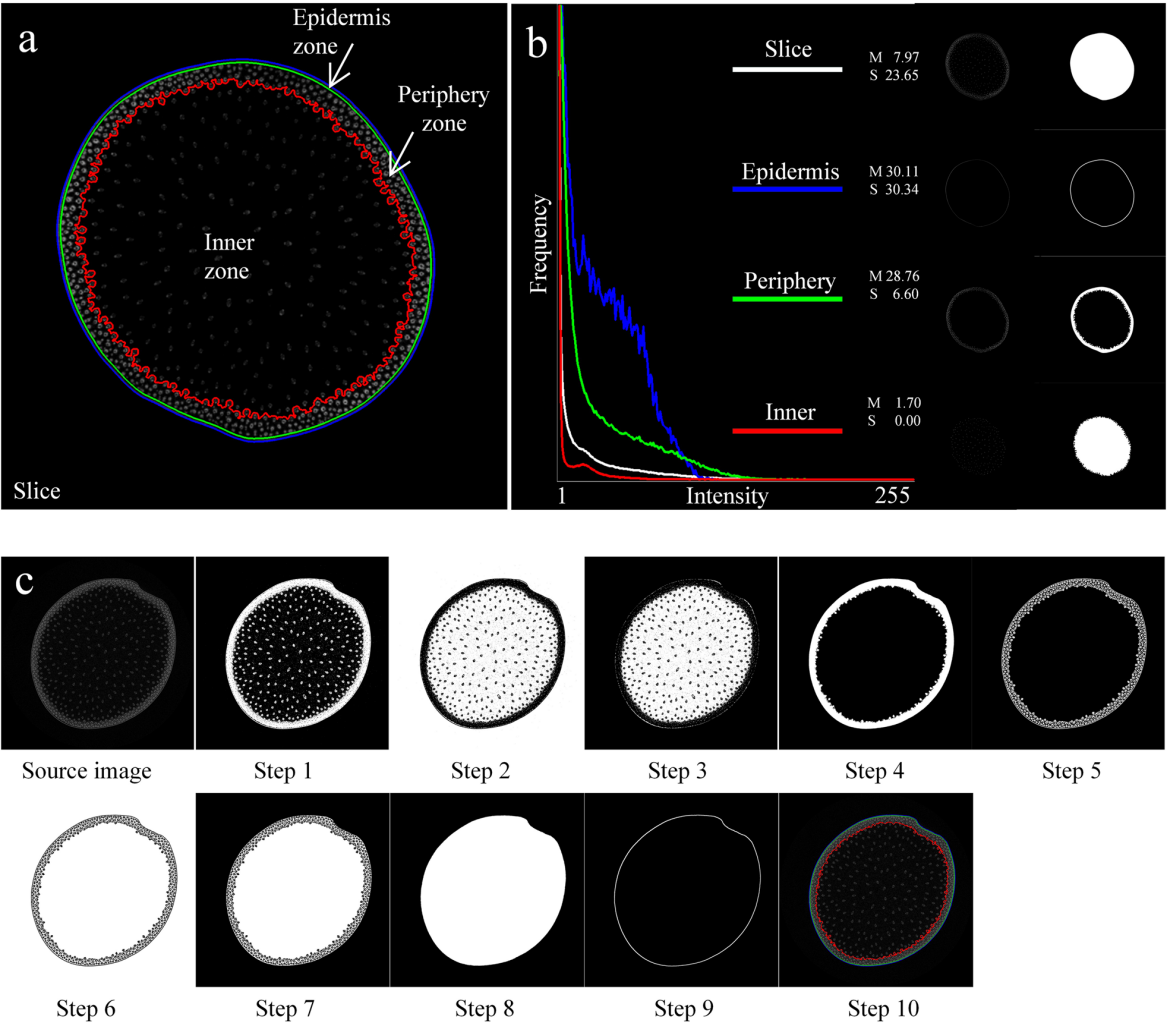
Schematic function zones of maize stem in a CT cross-section image. **a** The boundaries of the epidermis, periphery and inner zones. **b** The source and mask images of each individual zones, and the intensity and histogram results. **c** The detection pipeline of function zones

During the maize growth and development, the substance accumulation of maize stem manifests as the gradually increasing average HU values (i.e. intensity values) and dynamically changing distribution (i.e. connectivity relationship). According to the observable intensity and distribution differences between stem substances (shown as Fig. 1a), the entire stem slice could be reasonably divided into three function zones, i.e. epidermis zone, periphery zone, and inner zone. Figure 1b showed the source and mask images of slice, epidermis, periphery and inner zones, respectively. For each function zone, the intensity-based traits, such as intensity mean, standard variances and histogram, could be evaluated. In order to visualize histogram distribution in each function zone, background pixels which occupy the largest proportion were excluded from the histogram statistics. Thus, the value range of horizontal axis in the histogram was set as [1, 255], and the maximal value of vertical axis respectively corresponded to the maximal frequency of each function zone. Since the area (i.e. pixel number) of each function zone was different (e.g., the pixel number of the whole slice region was the largest, and the areas of periphery and epidermis zones were relatively smaller), thus maximal frequencies of histograms were greatly different. We plotted these histograms in the same coordinate system for visualization. Next, we depicted the detection pipeline of function zones in detail (Fig. 1c):

- Step 1: The source image was initially segmented using a fixed threshold value (that was responding to HU value of air).
- Step 2: Detected the outermost contour (Contour1), and judged whether the epidermis boundary was valid (an area ratio, i.e. 0.95, was used to evaluate the validation of epidermis boundary). Once epidermis boundary was valid, the generated image would be reversed by: Image = 255 − Image.
- Step 3: Detected the inner zone according to pixel connectivity, and determined the boundary between the inner and periphery zones (Contour2).
- Step 4: Extracted the periphery and epidermis zones according to Contour1 and Contour2.
- Step 5: Segmented the gray image of the periphery and epidermis zones using the adaptive threshold segmentation method, and resulted in epidermis and vascular bundles.
- Step 6: Reversed the mask image of Step 5 to make the epidermis zone and vascular bundles of the periphery zone as 0, and the cavity regions of the periphery zone as 255.
- Step 7: Removed the outside region of slice, thus only inner zone and cavity regions of the periphery zone were labeled as 255.
- Step 8: Performed closed operations to connect the cavity regions of the periphery zone together (the structure element size of morphological operations was estimated according to the biggest size of vascular bundles).
- Step 9: Extracted the outer boundary of the resulted mask image in Step 8 as Contour3, thus the epidermis zone could be determined by Contour1 and Contour3.
- Step 10: Contour1, Contour2 and Contour3 were combined to define the epidermis, periphery and inner zones.

In the algorithm pipeline above, function zones were detected and represented as a set of hierarchy contours which could be extracted from the substance image. However, boundaries of some stem samples were cracked, therefore it was necessary to identify whether the detected outer boundary of the slice was valid or not (Step 2). If the boundary was broken, the morphological operations would be used to repair the boundary contour. Since the boundary of stem sample was very close to convex shape, we could use a convex hull to fit the boundary and set its area as the maximum area value. And then, we detected the actual outer contour of stem sample, and took its area as the actual area. There was no doubt that the actual area should be less than the maximum area. Since this outer contour had to contain the most substances in the slice, 0.95 was an appropriate area ratio to evaluate and identify the availability of the detected contour. If the area ratio exceeded 0.95, the detected contour could be considered as the true boundary of stem sample, otherwise iterative morphological operations (i.e., dilate the binary image until its outermost contour was legal, and then eroded the contour with the same size) were used to repair the broken boundary of epidermis.

The boundary between the periphery and inner zone was detected in Step 3. The inner zone was entirely included within this boundary, and vascular bundles in the inner zone were fully independent from the surrounding tissues. Outside this boundary, vascular bundles usually connected into a whole or multiple individual region by the intermediate substances (parenchyma cells etc.).

In the most of cases, the epidermis and periphery zones were merged together by some vascular bundles. The boundary region between epidermis and periphery zones was ambiguous, thus a local adaptive threshold method based on mean was more appropriate for the separation between epidermis and periphery (Step 5). This classical method calculated the average value of all pixels in the given block region as a local optimal threshold instead of the specified threshold value. Therein, the block size was estimated as 31 according to experience and experiment.

The cavity regions of the periphery zone determined the boundary between the epidermis and periphery zones. Thus, the mask image of Step 5 was firstly reversed to make the cavity regions of the periphery zone as 255, and the epidermis zone and vascular bundles of the periphery zone as 0 (Step 6). Once the outside region of slice was removed, the cavity regions of the periphery zone would become the outermost regions of the slice (Step 7). To fill the vacancies of vascular bundles and smooth the boundary of cavity regions of periphery zone, closed operations were performed to merge cavity regions of the periphery zone together (Step 8). The structure element size of morphological operations was estimated according to the biggest size of vascular bundles, such as 15. Further, the outer boundary of cavity regions was extracted as Contour3 (Step 9). As a result, Contour1, Contour2 and Contour3 could be combined to determine the epidermis, periphery and inner zones (Step 10).

### Detection pipeline for vascular bundles

**Fig. 2:**
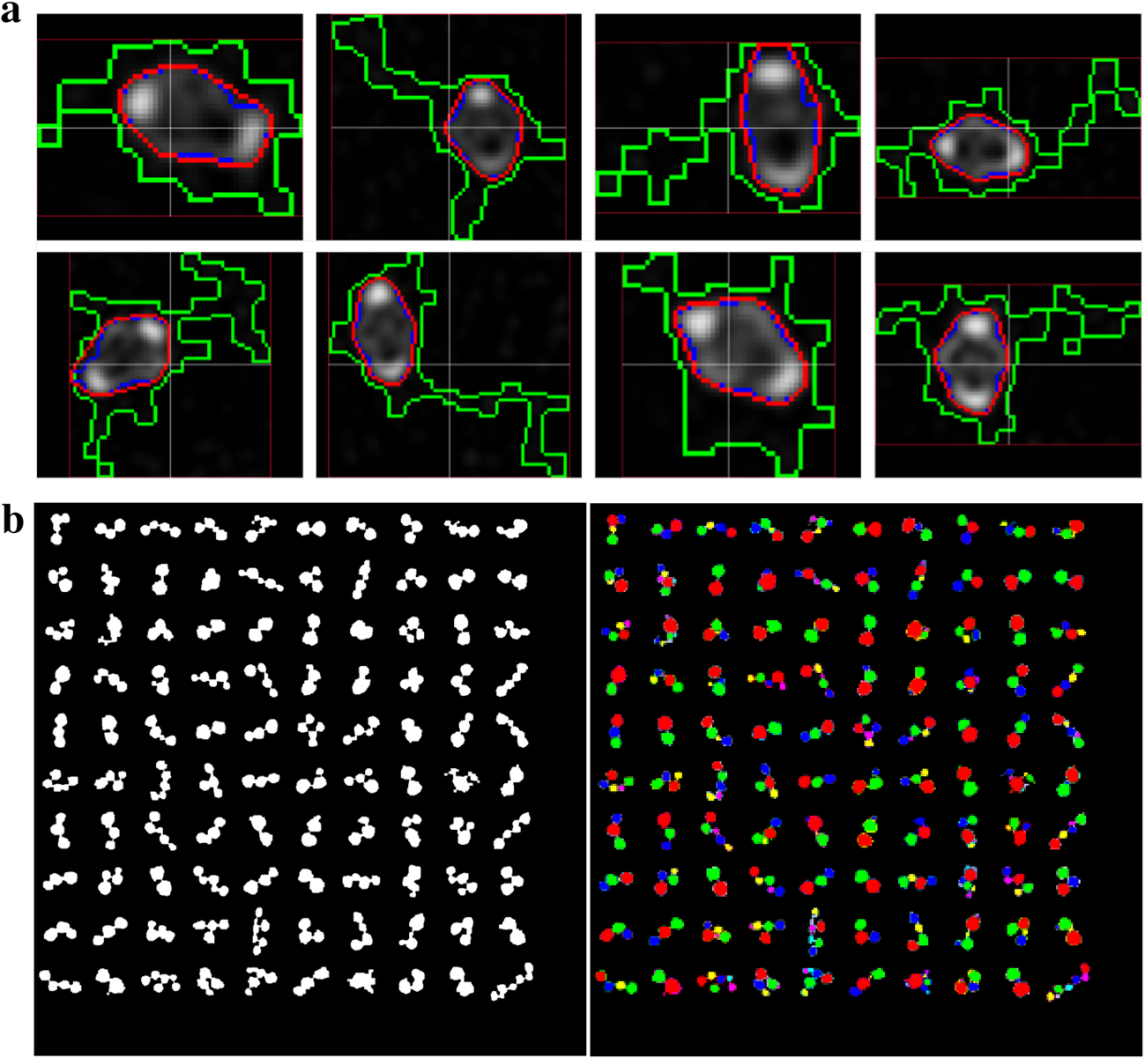
Detection and identification for vascular bundles. **a** Shape improvement for vascular bundles in the inner zone. **b** The object split of candidate regions for vascular bundles in the periphery zone

Vascular bundles in periphery and inner zones were distinctly different not only in size and morphology, but distribution and intensity. Vascular bundles of the periphery zones were densely distributed, and had a closer spacing distance, smaller area and more changeable intensity than ones in the inner zone. It was difficult to detect simultaneously vascular bundles of periphery and inner zones by the same method and parameters. Fortunately, the presented function zones provided valuable references for the segmentation and identification of vascular bundles. That is, image segmentation and object verification of vascular bundles could be respectively performed in the periphery and inner zones by the more adaptive methods. The detection pipeline of vascular bundles was described as follows:

For vascular bundles of the inner zone, the boundaries of vascular bundles were often equivocal due to the broken edge or less intensities. To protect the boundaries of vascular bundles in the inner zone, a fixed threshold value with 1 was used to result in redundant segmentation results that contained all boundaries of vascular bundles, but the size and shape were usually improper. Moreover, each segmented object in the inner zone could be taken as an individual candidate of vascular bundle. Therefore, Level set algorithm [26] was performed to track more appropriate boundary of these vascular bundles. Figure 2a demonstrated the shape improvement results of vascular bundles. Green contours were the redundant segmentation result of vascular bundles, blue contours indicated the improved shape by Level set method, and red contour represented the convex hulls of vascular bundles.

Vascular bundles in periphery zone were closely surrounded by the composites of lignin and cellulose, and were closely arranged. As a result, some candidates might contain several interconnected vascular bundles. Although these merged vascular bundles could be identified visually, the huge size difference and complex connectivity between vascular bundles were prone to decision-making ambiguity. In this study, an object splitting method based on shape features (inscribed circles) was used to subdivide the candidate object into several individual vascular bundles. This technique could generate a series of inscribed circles to fit and represent the shape of the target. That is, for a given object shape, the following operations were performed: (1) calculated the maximum inscribed circle of the given object, (2) deleted this inscribed circle region from the input image, and (3) the remaining area as new input image was performed step (1) and (2), until the radius of the new inscribed circle was less than a small threshold, such as 3. As a result, the given object was represented as a serial of inscribed circles with different size. As an example, 100 candidate regions were respectively collected from the previous results, and then were split into individual vascular bundles by the presented method, as shown in Fig. 2b. Each candidate region with complex shape was split into more than two individual regions.

### Phenotyping for vascular bundles

Contour representation was an effective and simple shape descriptor for the entire slice, function zones, and vascular bundles. Contour could be directly used to calculate the geometry-based traits. And the image region surrounded by this contour also could be used to calculate the intensity-based traits, such as mean intensity and variance. Moreover, the contour center represented the true object position, thus it was also used to calculate the distribution-based traits.

### Layer-related traits of vascular bundles

**Fig. 3:**
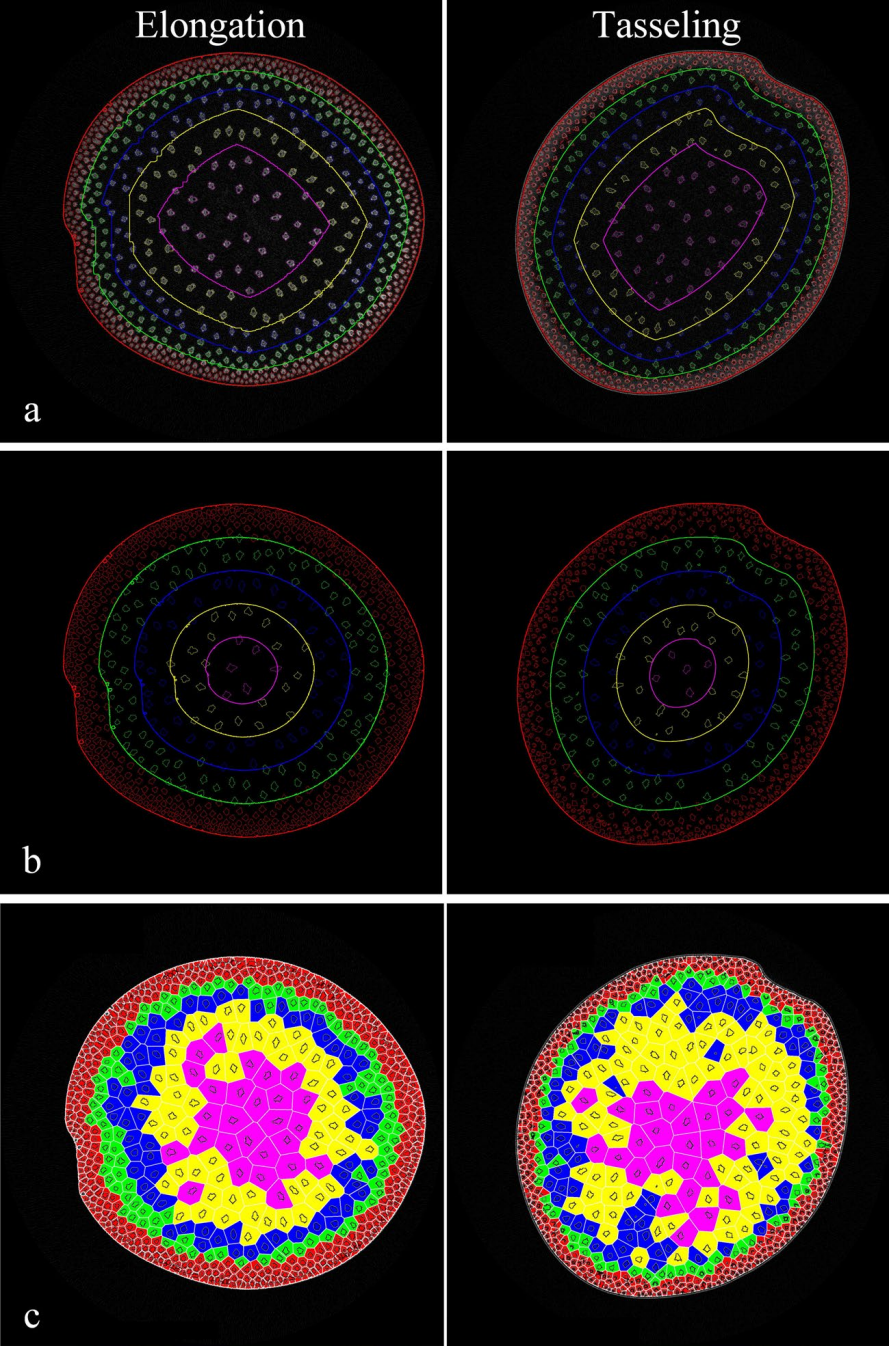
Distribution-related traits of vascular bundles. **a** Layer scheme with equal-area. **b** Layer scheme with equal-distance. **c** Voronoi diagrams of vascular bundles

**Fig. 4:**
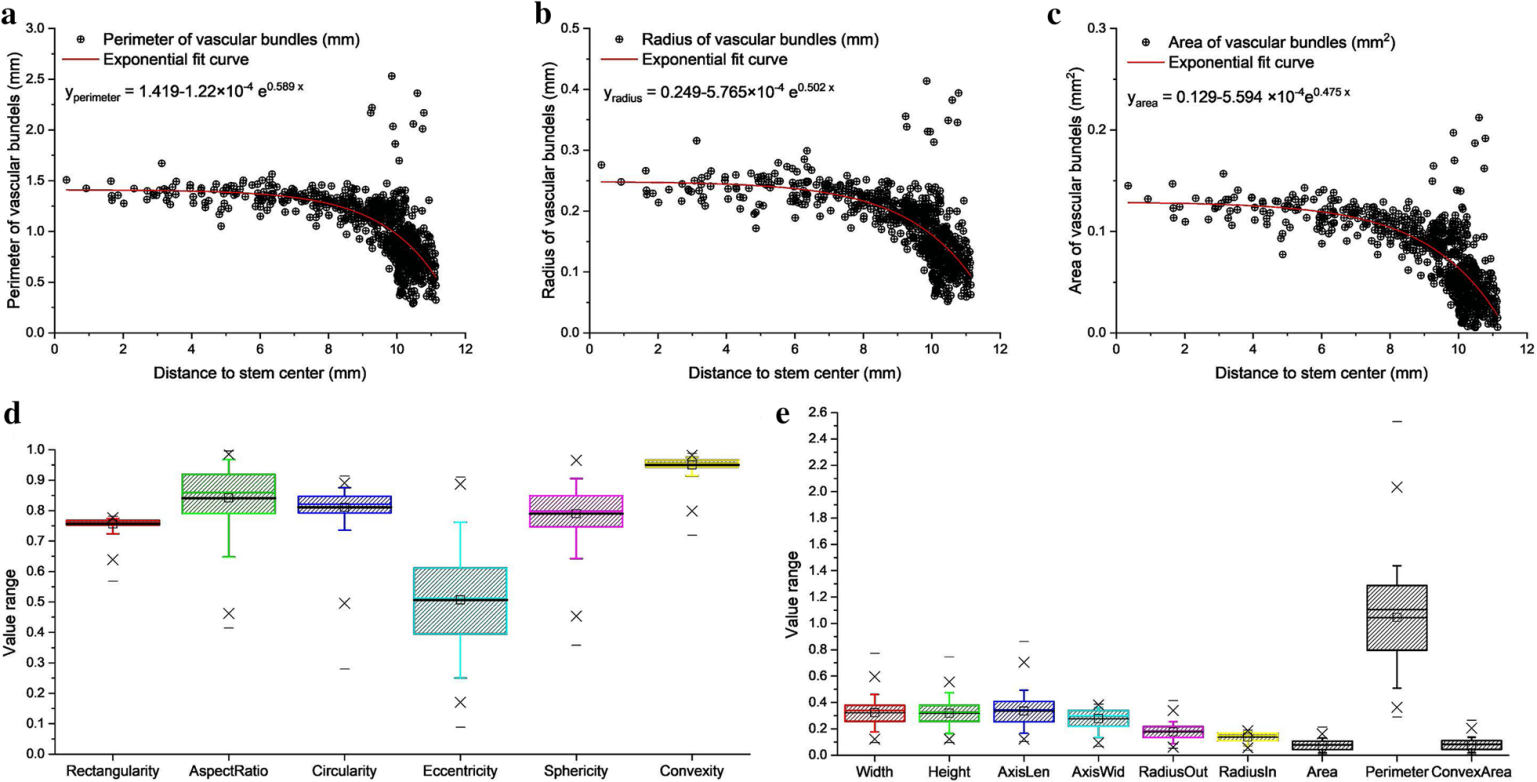
The geometry-related and distribution-related traits of vascular bundles. **a**–**c** The relationship between geometry-related traits (perimeter, radius, area) of vascular bundles and the distance (from the center of vascular bundle to stem center). **d** The value ranges of 6 dimensionless geometry-related traits of vascular bundles. **e** The value ranges of 9 dimensional geometry-related traits of vascular bundles

To quantify the distribution of vascular bundles in the entire slice, two layer schemes have been implemented to divide the slice into several ring regions, as described in the literature [24]. Several geometrical contours related with the stem center were generated to divide the maize stem into equal-distance (ED) or equal-area (EA) regions, as shown in Figs. 3a and 4b. Delaunay triangulation techniques were used to generate graph representation (such as triangles and patches) of vascular bundles by taking the center points of all vascular bundles as initial nodes, and the inner boundary of epidermis as a boundary constraint. According to the areas of vascular bundles, K-Mean clustering technique was performed to classify these graphs into several categories labeled with different pseudo-colors (shown as Fig. 3c).

### Implementation

To quantify micro phenotypes of maize stems for large-scale samples from different growth stages is still a challenge. Here, we developed a set of automated phenotyping pipelines to handle with a large-scale CT images in a batch processing style. All image processing and analysis steps were conducted in Visual studio C++ and OpenCV.

Based on uniform contour representation, almost all of the objects detected from the CT image were represented as contours, and each contour could be represented as a set of points. Thus, the data structure could be stored or loaded as a local file (VBF) by serialization mechanism. This file could not only record all semantic information parsed by the image pipeline, such as vascular bundles, layers and function zones, but be conducted the further statistic analysis of the slice and vascular bundles. As a result, the presented μPhenotyping pipelines can automatically process a large scale of samples, and well organize the related traits of the stem and vascular bundles together for statistical analysis.

## Results

### Quantification of stem phenotypes

Table 1 listed five categories of phenotypic traits of vascular bundles, including intensity-related, geometry-related, distribution-related, layer-related, and growth-related traits. In this study, the geometry-based traits were classified into dimensional and dimensionless parameters. Six dimensionless parameters (rectangularity, aspect ratio, circularity, eccentricity, sphericity and convexity) were irrelevant to the size and orientation of contour shape, and nine dimensional parameters (i.e. width, height, main axis length, main axis width, circumcircle radius, inscribed circle radius, area, perimeter, and convex hull area) were relevant to the size and orientation of contour shape, with mm or mm^2^ units.

**Table 1:** List of five categories 28 phenotypic traits of stem obtained by automated image processing pipeline

Phenotypic types	Description	Units
Intensity-based	Average intensity (AI)	Gray-level
Geometry-based (dimensional)	Width (W)	mm
	Height (H)	mm
	Main axis length (MAL)	mm
	Main axis width (MAW)	mm
	Circumcircle radius (CR)	mm
	Inscribed circle radius (ICR)	mm
	Area (A)	mm^2^
	Perimeter (P)	mm
	Convex hull area (CHA)	mm^2^
Geometry-based (dimensionless)	Rectangularity (RA), ${\text{RA}} = \frac{{\text{A}}}{{{\text{MAL}} \times {\text{MAW}}}}$	–
	Aspect ratio (AR), ${\text{AR}} = \frac{MAW}{{MAL}}$	–
	Circularity (CIR), ${\text{CIR}} = \frac{4\pi \cdot A}{{P^{2} }}$	–
	Eccentricity (ECC), ${\text{ECC}} = \frac{{\sqrt {MAL^{2} - MAW^{2} } }}{MAL}$	–
	Sphericity (SPH), ${\text{SPH}} = \frac{{{\text{ICR}}}}{{{\text{CR}}}}$	–
	Convexity (CV), ${\text{CV}} = \frac{A}{CHA}$	–
Distribution-based	Distance from vascular bundle to stem center (DC)	mm
Layer-based (equal-distance, equal-area)	Area of each layer (AEL)	mm^2^
	Number of vascular bundles in each layer (NVBEL)	–
	Area of vascular bundles in each layer (AVBEL)	mm^2^
	Voronoi area of vascular bundle in each layer (VAVBEL)	mm^2^
Growth-based (epidermis, periphery, inner)	Area of each function zone (AEFZ)	mm^2^
	Number of vascular bundles in periphery zone (NVBPZ)	–
	Number of vascular bundles in inner zone (NVBIZ)	–
	Area of vascular bundles in periphery zone (AVBPZ)	mm^2^
	Area of vascular bundles in inner zone (AVBIZ)	mm^2^
	Voronoi area of vascular bundle in periphery zone (VAVPZ)	mm^2^
	Voronoi area of vascular bundle in inner zone (VAVIZ)	mm^2^

The geometry-related traits of vascular bundles were strongly related to their position relative to the stem center. Thus, the distance from vascular bundle to the stem center was taken as a distribution descriptor. Figure 4a–c showed the relationship between geometry-related traits (perimeter, radius and area) of candidates and their positions, respectively. A serial of exponential curves were fitted to indicate the distribution of candidates, as follows:

$$\left\{ \begin{aligned} & y_{{{\text{perimeter}}}} = 1.419 - 1.22 \times 10^{ - 4} e^{0.589 x} \\ &y_{{{\text{radius}}}} = 0.249 - 5.765 \times 10^{ - 4} e^{0.502x}\\ & y_{{{\text{area}}}} = 0.129 - 5.594 \times 10^{ - 4} e^{0.475 x} \\ \end{aligned} \right.$$

Therein, *x* indicates the distance from the center of candidates to the stem center, and *y* indicates the shape features of candidates, i.e. radius, area and perimeter. As far away from the slice center, the number of candidates rapidly increased, and all dimensional geometry-based traits rapidly reduced exponentially. The most candidates assembled in the outer layer of the slice (about 10 mm from the slice center), however dozens of candidates in the farthest region were obviously deviated from the fit curves. Thus, these candidates were likely to contain multiple vascular bundles. Figure 4d, e showed the value ranges of geometry-related traits of candidates. As a result, these geometry-related and distribution-related traits of candidates provided reasonable grounds to determine whether they represented valid vascular bundles or not. Actually, these traits could be utilized to build machine learning model (such as support vector machine, SVM) and embedded into the detection pipeline for vascular bundles.

**Fig. 5:**
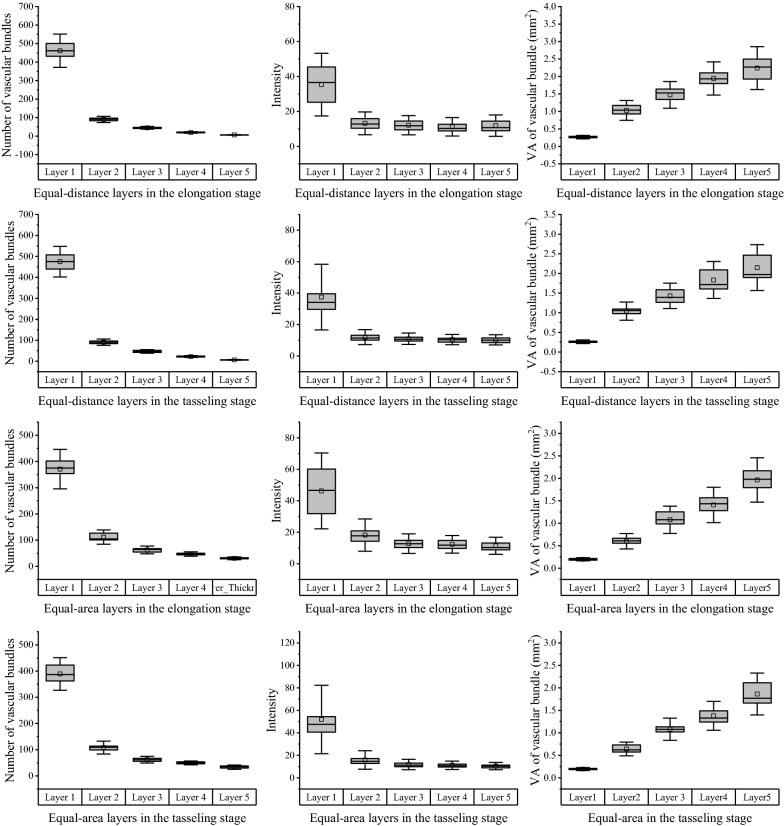
Analysis of stem microscopic phenotypic traits (vascular bundle number, intensity, and vascular bundle voronoi area) in EA and ED layers at elongation and tasseling stages

Moreover, to quantify the distribution of vascular bundles in the entire slice, two layer schemes were implemented to divide the slice into several ring regions, i.e. equal-distance (ED) or equal-area (EA) regions (as shown as Fig. 3a, b). In the voronoi diagram shown in Fig. 3c, each patch corresponded to an individual vascular bundle, and indicated the exclusive growth space of this vascular bundle. Starting from the stem center to the inner epidermis boundary, the slice was divided into five adjacent layers, and marked as layer 1–5. The average number of vascular bundles from the 1st to 4th layers moderately increased but a huge increase came up in the 5th layer. Average intensity of different layer showed the same variation tendency as the number of vascular bundles. On the contrary, voronoi areas of vascular bundles were demonstrated similarly linear declining trend from 1st to 5th in two growth stages. As shown in Fig. 5a, b, equal-distance and equal-area schemes described the similar distribution characteristics of vascular bundles at the elongation and tasseling stages. Compared with distribution-related traits based on the distance, the layer schemes can actually be regarded as distribution-related traits based on layer areas of vascular bundles.

### Function zone distributions at elongation and tasseling stages

**Fig. 6:**
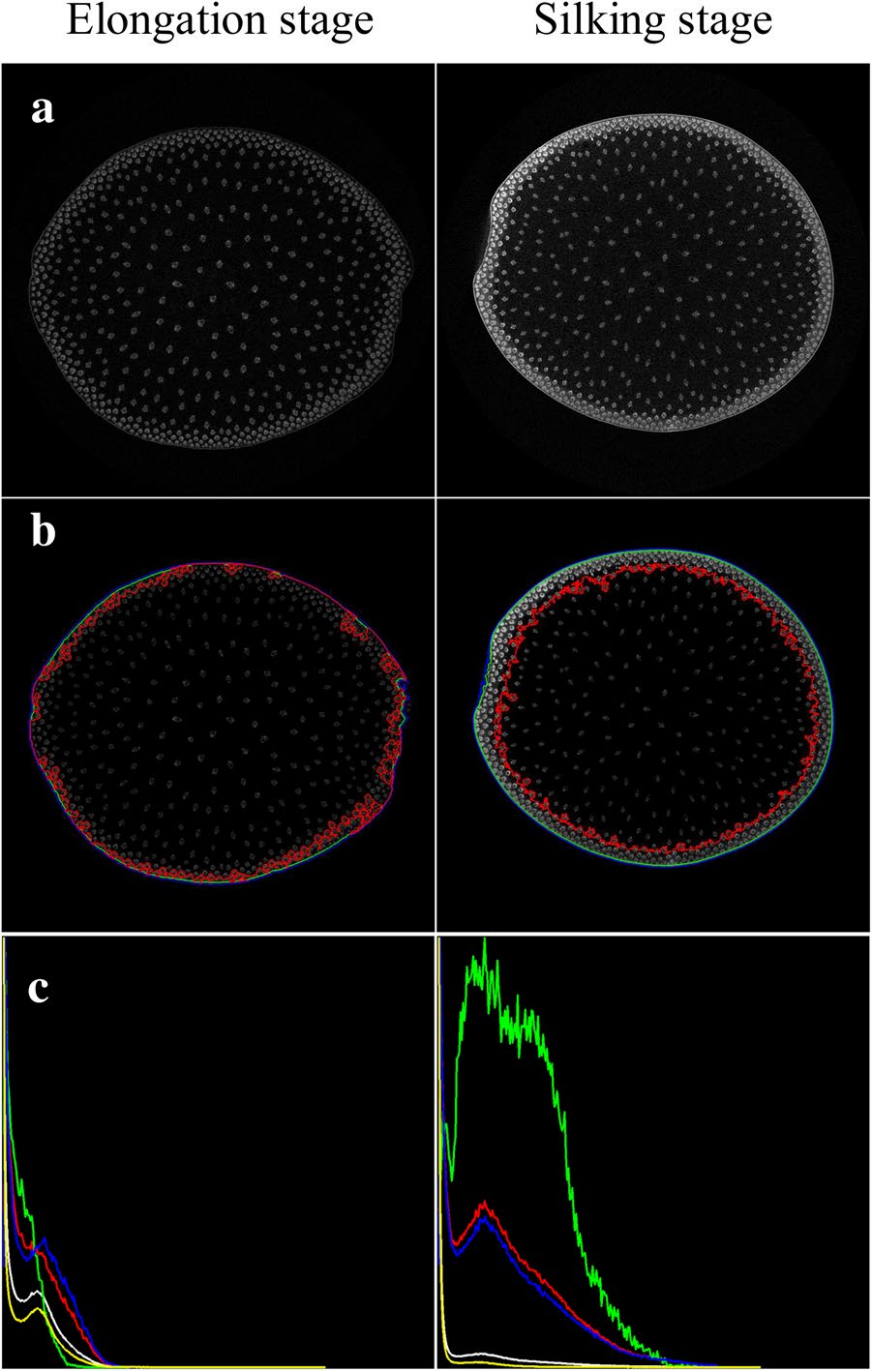
Function zone distributions of maize stem (Jingke665) at the elongation and tasseling stage. **a** The source images of stem at elongation and tasseling stages. **b** Function zone distributions of maize stem at elongation and tasseling stages. **c** Histograms of different zones, the whole slice (white), epidermis zone (green), periphery zone (blue), inner area (yellow), and the combination of the epidermis and periphery zones (red)

Phenotypic traits of function zones provided direct indicators to evaluate the specific physiological status of maize stem at elongation and tasseling stages. For the different growth periods, i.e. elongation and tasseling stages, correspond to the vegetative and reproductive stages respectively, the intensity and structure of the maize stem and which vascular bundles were much different (Fig. 6a). Accurate detection of the function zones was the precondition of the segmentation and verification of vascular bundles. The function zones of the same maize cultivar (Jingke665) at elongation and tasseling stages were shown in Fig. 6b. Three contours respectively represented the boundaries of function zones, i.e. the outer epidermis contour (blue), the outer periphery contour (cyan) and the inner periphery contour (red). From the elongation to tasseling stages, maize plants grew rapidly along with greater demand for nutrients and water, and morphological characteristics of vascular bundles changed dramatically accordingly. In Fig. 6c, the histogram distributions of the whole slice (white), epidermis zone (green), periphery zone (blue), inner zone (yellow), and surrounding region (red, considering the epidermis and periphery zones together) revealed the substance distribution within maize stem. The changes of histograms also revealed the growth and development characteristics of maize stem. From the elongation to tasseling stages, the substances of maize stem rapidly accumulated in the epidermis and the periphery zones, especially the epidermis zone. The huge intensity and distribution differences between periphery and inner zones indicated phenotypic traits of function zones could effectively describe the dynamic growth and substance accumulation of maize stem.

### Growth-related traits of the slice and epidermis zone at different growth stages

**Fig. 7:**
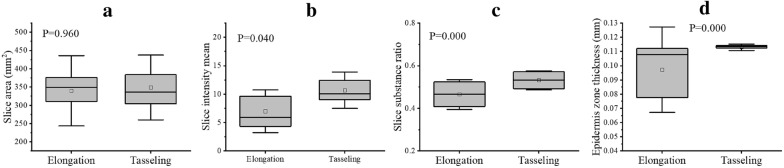
Growth-related traits of the slice and epidermis zone at the elongation and tasseling stages. **a** Slice area of stem. **b** Slice intensity of stem. **c** Slice substance ratio of stem. **d** Epidermis thickness of stem

In this study, the slice areas of the stem samples in different growth stages were very similar, the average slice area of 20 varieties from the elongation to tasseling stages was 339.8 mm^2^ and 348.9 mm^2^. However, the intensity and substance ratio of maize stems were demonstrated significant differences from the elongation to tasseling stages (Fig. 7b, c). The difference in slice intensity of maize stem was almost twice as much from the elongation to tasseling stages, and the substance ratio (i.e. the ratio of non-zero pixels to all slice pixels) increased significantly. Moreover, the epidermis thickness was one of the most important structural indicators to describe the growth state of maize stem. At the elongation stage, the epidermis thickness had a large variance related with cultivar attributes, but became stable at the tasseling stage (Fig. 7d). It maybe means the epidermis rapidly grows at the elongation stage, and establishes morphogenesis at the tasseling stage.

### Growth-related traits of periphery and inner zones in different growth stages

From the elongation to tasseling stages, not only substance contents of the maize stem but the geometry and distribution of vascular bundles changed greatly. The function zones of maize stem quantified these differences in a new viewpoint, thus new growth-related traits based on function zones were calculated and used for more refined evaluation of the dynamic growth of maize stems.

**Fig. 8:**
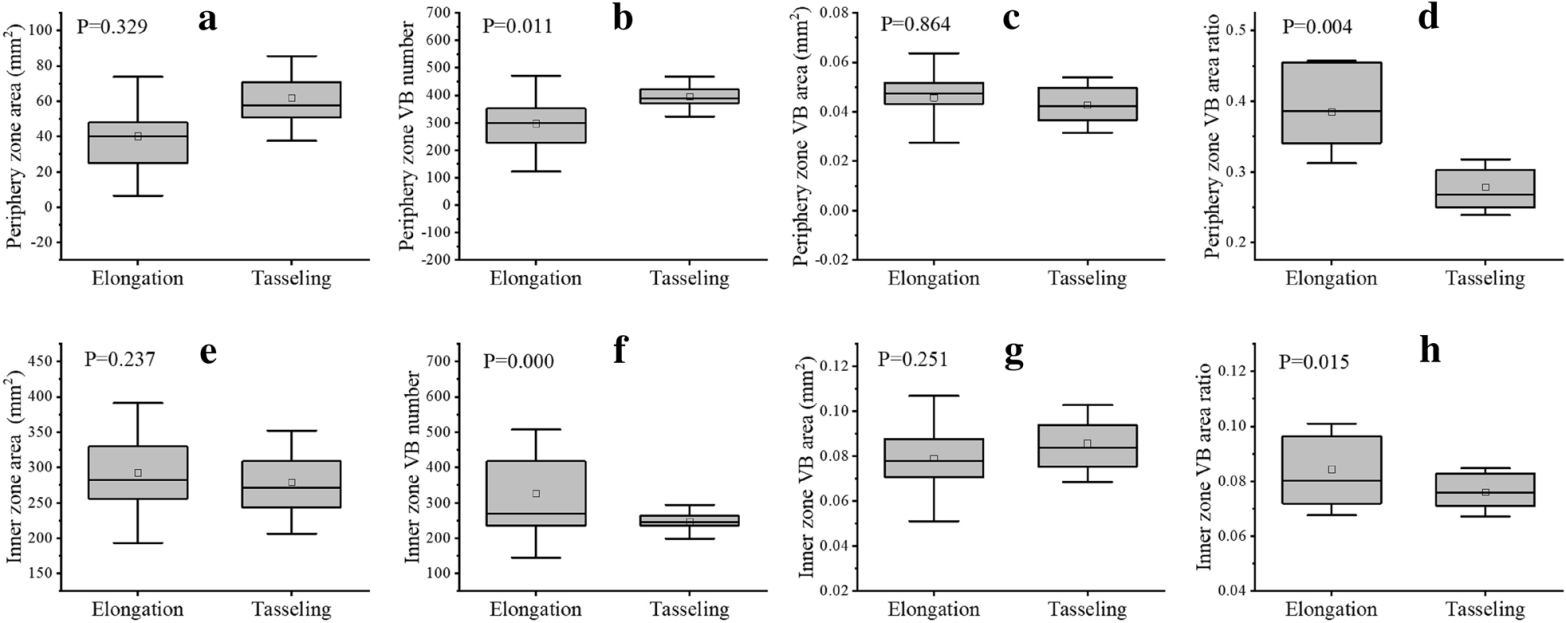
Growth-related traits of the periphery and inner zones at the elongation and tasseling stages. **a**–**d** Phenotypic traits of periphery zone (**a** zone area, **b** vascular bundle number, **c** vascular bundle area, and **d** area ratio of vascular bundle). **e**–**h** Phenotypic traits of inner zone (**e** zone area, **f** vascular bundle number, **g** vascular bundle area, and **h** area ratio of vascular bundle)

At the elongation stage, the average area of inner zone for 20 different varieties was 292.2 mm^2^, which was about 7 times than that of periphery zone (Fig. 8a, e), but the average number of vascular bundles of two different zones was quite similar, 325.8 and 296.5 respectively (Fig. 8b, f). Moreover, the average area (Fig. 8c, g) of vascular bundles in the inner zone were much bigger than that in the periphery zone. At the tasseling stage, the average area of inner zone for 20 different varieties was 279.1 mm^2^, which was about 5 times than that of periphery zone (Fig. 8a, e), but the average number of vascular bundles in the inner zone was only half of that in the periphery zone, which were 246.5 and 395.1 respectively (Fig. 8b, f). The average area of vascular bundles in the inner zone was about twice as much as one in the periphery zone (Fig. 8c, g).

From the elongation to tasseling stages, the area of periphery zone maintained progressive increase, but the inner zone decreased in a relatively lower speed (Fig. 8a, e). The number of vascular bundles in the periphery zone rapidly increased but had a slightly decreased in the inner zone, simultaneously (Fig. 8b, f). On the contrary, the average area of vascular bundles demonstrated different changes. The average area of vascular bundle in inner zone showed a faster growth than that in the periphery zone (Fig. 8c, g). This might be closely related to the increase of material transport capacity of stem vascular bundle during the reproductive growth stage.

Further, we combined vascular bundle number, vascular bundle area, and function zone area to calculate the area ratio of vascular bundles in the periphery and inner zones, respectively (Fig. 8d, h). At the two stages, the area ratio of vascular bundles in the periphery zone was much larger than that in the inner zone. And from the elongation to tasseling stages, the area ratio of vascular bundles in the inner zone had not changed much, but that in periphery zone decreased significantly. Experimental results above showed that the periphery and inner zone division demonstrated more refined growth-related traits of vascular bundles, such as the vascular bundle number, area, and distribution traits. Moreover, the vascular bundle number and area ratio of vascular bundles might be an effective indicator to evaluate the dynamic growth of maize stem and cultivar differences.

## Discussion

In earlier work, a large number of methods have been developed for the detection of micro anatomical characteristics of cross-section stem, to meet the need for large-scale measurements of stem anatomy features [21–25, 27]. In spite of improved measurement efficiency of vascular bundle, the robustness and general applicability of these tools need to be improved. Here, the results of μPhenotyping pipelines for maize stem were well be organized and serialized as a VBF file. Through this pipeline, contour representations of the slice, function zones, layers, and vascular bundles, provided uniform analysis process to output lots of traits, such as intensity-related, geometry-related, distribution-related and growth-related traits.

**Fig. 9:**
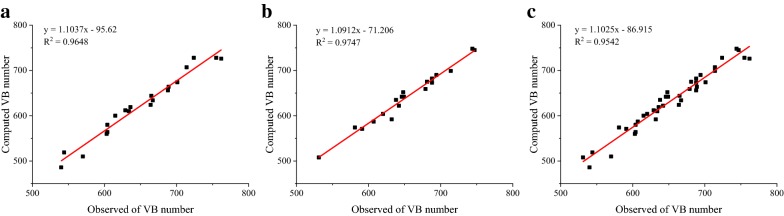
Counting accuracy comparison of vascular bundles numbers by the presented method and manual counting using stem samples from the elongation stage (**a**), stem samples from tasseling stage (**b**), and stem samples from two stages (**c**)

In order to evaluate the counting accuracy of vascular bundles, 40 CT images (i.e. 20 different varieties, and two growth stages) were randomly selected for manual counting. Counting results of vascular bundles by the manual investigation and presented method were shown in Fig. 9. The coefficient of determination (R^2^) of the observed and computed values at the tasseling stage (Fig. 9b) was slightly higher than that at the elongation stage (Fig. 9a), and R^2^ using all sample from two stages (Fig. 9c) was lowest but reached 0.9542. It indicated the presented method could effectively detect vascular bundles both at the elongation and tasseling stages, even more propitious to mature growth stages owing to bigger intensity of stem substances and segmentation schemes based on function zones. Moreover, μPhenotyping pipelines for maize stem were organized into a batch processing, and the average computation time was about 20 s per image.

Function zones of maize stem play a crucially important role not only for the detection pipeline but for the phenotyping pipeline of vascular bundles. The periphery and inner zones are sensitive to growth and development status of maize stem, thus it is effective to identify growth-related phenotypic traits, such as estimation of the biomass, maturity degree, bio-mechanical properties etc. The dynamic development of maize stems and vascular bundles from the elongation to tasseling stages can be well described by growth-related traits of function zones. These traits are highly valuable for revealing the development mechanism and understanding the relationship between anatomical structure and physiological function of vascular bundles [28]. Efficient and accurate µPhenotyping technology is opening the door to studies that integrate vascular bundle functional genomics with phenomics, to provide novel insights in development and functions of the maize vascular system. Future wok therefore will focus on quantification of growth-related traits of maize stem in more growth stages to reveal the successive and dynamic growth and development features.

## Conclusions

We have presented a set of image-based μPhenotyping pipelines to quantify maize stem images acquired using μCT scanning technology. The robustness and accuracy of the presented method are evaluated using stem samples from different maize varieties and growth stages. Compared with the previous methods, a dominant advantage of this method was its more abundant phenotypic indicators and much wider application for different growth stages. And growth-related traits of the slice, epidermis, periphery and inner zones provided novel indicators to describe the dynamic growth of maize stem. Using these rich phenotypic characteristics, it is highly crucial for understanding the relationship between stem phenomics and genomics.

## Supplementary information

**Additional file 1.** The pseudo-codes of related algorithms and data structure.

## Data Availability

All data generated or analysed during this study are included in this published article and in Additional file 1.

## Abbreviations

AI: average intensity
W: width (mm)
H: height (mm)
MAL: main axis length (mm)
MAW: main axis width (mm)
CR: circumcircle radius (mm)
ICR: inscribed circle radius (mm)
A: area (mm^2^)
P: perimeter (mm)
CHA: convex hull area (mm^2^)
RA: rectangularity
AR: aspect ratio
CIR: circularity
ECC: eccentricity
SPH: sphericity
CV: convexity
DC: distance from vascular bundle to stem center (mm)
AEL: area of each layer (mm^2^)
NVBEL: number of vascular bundles in each layer
AVBEL: area of vascular bundles in each layer (mm^2^)
VAVBEL: voronoi area of vascular bundle in each layer (mm^2^)
AEFZ: area of each function zone (mm^2^)
NVBPZ: number of vascular bundles in periphery zone
NVBIZ: number of vascular bundles in inner zone
AVBPZ: area of vascular bundles in periphery zone (mm^2^)
AVBIZ: area of vascular bundles in inner zone (mm^2^)
VAVPZ: voronoi area of vascular bundle in periphery zone (mm^2^)
VAVIZ: voronoi area of vascular bundle in inner zone (mm^2^)
ED: equal-distance
EA: equal-area
SVM: support vector machine
