# Supplementary material for: Phenotyping analysis of maize stem using micro-computed tomography at the elongation and tasseling stages
Source: Plant Methods. 2020 Jan 4;16:2. doi: 10.1186/s13007-019-0549-y (PMC6942302; doi:10.1186/s13007-019-0549-y)
Supplement: Supplementary file 1 — Additional file 1. The pseudo-codes of related algorithms and data structure. [file 13007_2019_549_MOESM1_ESM.doc]

We provide pseudo-codes and notations of related data structure and algorithms in this paper.

1. **Data structure of VBF**

typedef vector<cv::Point> CONTOURPTS; // contour

typedef vector< CONTOURPTS > CONTOURS; // contour vector

typedef vector< CONTOURPTS* > PCONTOURS; // pointer of contour

class AFX_CLASS_EXPORT duStemVascularBundle : public CObject

{

public:

duStemVascularBundle();

~duStemVascularBundle();

DECLARE_SERIAL(duStemVascularBundle) // serialization mechanism

virtual void Serialize(CArchive& ar); // serialization mechanism

BOOL SaveToFile(CString strFile); // store data to VBF file

BOOL LoadFromFile(CString strFile); // load data from VBF file

CString strImage; // source image

int nWidth; // image with

int nHeight;// image height

double m_dPixelSize; // pixel size

// function zones

CONTOURPTS conOuterEpidermis; // outer contour of epidermis, Contour1

CONTOURS consPeriphery; // contours between inner and periphery zones, Contour2

CONTOURPTS conInnerEpidermis; // contour between epidermis and periphery zones, Contour3

// layers and voronoi data

CONTOURS vecContourLevels; // layers

std::vector<double> vecThick_layer; // thickness of each layer

std::vector<double> vecArea_layer; // area of each layer

CONTOURS vec_LayerLevels_Area; // contours with equal-area layers

CONTOURS vec_LayerLevels_Dist; // contours with equal-distance layers

std::vector<LABEL_VORONOI> vec_voronoi_layers; // for voronoi analysis based on K-Mean

std::vector<duSingleVB*> vecVascularBundles; // each vascular bundle was extracted, store for statistical analysis

// vascular bundles structues in zones

CONTOURS cons_innerzone_vbb;

CONTOURS cons_peripheryzone_vbb;

CONTOURS cons_refined_vbb;

};

1. **Level set method was implemented in C++.**
2. Call interface

CONTOURS consM; // store improved contours

std::vector<Mat> vecSIIter;

int nNumofIter = duLevelSet::DetectObjects(

srcImage, // source image

consIn, // input contours,

consM, // improved contours

spp.nLevelSetIterationNum, // iterator number

vecSIIter // intermediate results

);

1. Source code of level set method (.h and .cpp files)

//.h file

#pragma once

#include <vector>

#include <opencv2/opencv.hpp>

#include "duContours2.h"

using namespace cv;

using namespace std;

namespace MCCM // an ongoing phenotyping project

{

class AFX_CLASS_EXPORT duLevelSet

{

public:

duLevelSet();

~duLevelSet();

// input initial contours and iteration number, and return improved contours

static int DetectObjects(

const Mat& srcImage,

const CONTOURS& consBoundary,

CONTOURS& cons_out,

int nIterNum,

std::vector<Mat>& vecSIIter

);

// Initialization level set

void initializePhi(

Mat img,

int iterNum,

CONTOURS contours

);

// Evolution and record evolution steps

int EVolution(std::vector<Mat>& vecSIIter);

// base algorithm parameter

int m_iterNum; // iter number

float m_lambda1; // Global Term Coefficient

float m_nu; // Length Constraint Coefficient,ν

float m_mu; //Penalty coefficient, μ

float m_timestep; //Evolution step length, δt

float m_epsilon; //Regularized parameters, ε

int m_iCol; // image width

int m_iRow; // iamge height

int m_depth; // Level Set Data Depth

float m_FGValue; // Foreground value

float m_BKValue; // Background value

// process data

Mat m_mImage; // source image, Grey

Mat m_mPhi; // Levelset：φ

// output.

vector<vector<Point> > cons_out;

protected:

Mat m_mDirac; // Level Sets after Dirac Processing:δ(φ)

Mat m_mHeaviside; // Level Sets after Heidegger Function Processing:Н(φ)

Mat m_mCurv; // Curvature of level set:κ=div(▽φ/|▽φ|)

Mat m_mK; // Penalty term convolution kernel

Mat m_mPenalize; // In the penalties: ▽<sup>2</sup>φ

void Dirac(); //Dirac function

void Heaviside(); //Heidegger function

void Curvature(); //curvature

void BinaryFit(); //Calculate foreground and background values

};

}

//.cpp file

#include "stdafx.h"

#include "duLevelSet.h"

#include<iostream>

#include<opencv.hpp>

using namespace std;

using namespace cv;

namespace MCCM // name space in our project

{

Mat showIMG; // intermediate image

duLevelSet::duLevelSet()

{

// default algorithm parameters

m_iterNum = 300;

m_lambda1 = 1;

m_nu = 65;

m_mu = 1.0;

m_timestep = 0.1;

m_epsilon = 1.0;

}

duLevelSet::~duLevelSet()

{

}

int duLevelSet::DetectObjects(

const Mat& srcImage,

const CONTOURS& consBoundary,

CONTOURS& cons_out,

int nIterNum,

std::vector<Mat>& vecSIIter

)

{

USES_CONVERSION;

CONTOURPTS pts;

for (int i = 0; i < consBoundary.size(); i++)

{

std::copy(consBoundary[i].begin(), consBoundary[i].end(), std::back_inserter(pts));

}

Rect bbox = cv::boundingRect(pts);

Mat greyROI = Mat::zeros(bbox.size(), CV_8UC1);

if (srcImage.type() == CV_8UC1)

srcImage(bbox).copyTo(greyROI);

else

{

Mat grey = Mat::zeros(srcImage.size(), CV_8UC1);

cv::cvtColor(srcImage, grey, CV_BGR2GRAY);

grey(bbox).copyTo(greyROI);

}

CONTOURS cons_sub;

duContours2::CONTOURS_PAN(consBoundary, Point(-bbox.x, -bbox.y), cons_sub);

Mat maskROI = Mat::zeros(bbox.size(), CV_8UC1);

Mat greyROI2 = Mat::zeros(bbox.size(), CV_8UC1);

cv::drawContours(maskROI, cons_sub, -1, 255, -1);

cv::bitwise_and(greyROI, greyROI, greyROI2, maskROI);

// call level set method

duLevelSet ls;

ls.initializePhi(

greyROI2,

nIterNum,

cons_sub

);

int nNumOfIter = ls.EVolution(vecSIIter);

duContours2::CONTOURS_PAN(ls.cons_out, Point(bbox.x, bbox.y), cons_out); // translate contours

return nNumOfIter;

}

void duLevelSet::initializePhi(Mat img, int iterNum, CONTOURS contours)

{

m_iterNum = iterNum;

if (img.type() == CV_8UC3)

cvtColor(img, m_mImage, CV_BGR2GRAY);

else

{

m_mImage = Mat::zeros(img.size(), CV_8UC1);

img.copyTo(m_mImage);

}

m_iCol = img.cols;

m_iRow = img.rows;

m_depth = CV_32FC1;

m_mPhi = Mat::zeros(m_iRow, m_iCol, m_depth);

m_mDirac = Mat::zeros(m_iRow, m_iCol, m_depth);

m_mHeaviside = Mat::zeros(m_iRow, m_iCol, m_depth);

m_mK = (Mat_<float>(3, 3) << 0.5, 1, 0.5,

1, -6, 1,

0.5, 1, 0.5);

int c = 5;

// according to mask

Mat mask = Mat::zeros(img.size(), CV_8UC1);

cv::drawContours(mask, contours, -1, 255, -1);

m_mPhi.setTo(-c);

m_mPhi.setTo(c, mask);

}

void duLevelSet::Dirac()

{

float k1 = m_epsilon / CV_PI;

float k2 = m_epsilon*m_epsilon;

for (int i = 0; i < m_iRow; i++)

{

float *prtDirac = &(m_mDirac.at<float>(i, 0));

float *prtPhi = &(m_mPhi.at<float>(i, 0));

for (int j = 0; j < m_iCol; j++)

{

float *prtPhi = &(m_mPhi.at<float>(i, 0));

prtDirac[j] = k1 / (k2 + prtPhi[j] * prtPhi[j]);

}

}

}

void duLevelSet::Heaviside()

{

float k3 = 2 / CV_PI;

for (int i = 0; i < m_iRow; i++)

{

float *prtHeaviside = (float *)m_mHeaviside.ptr(i);

float *prtPhi = (float *)m_mPhi.ptr(i);

for (int j = 0; j < m_iCol; j++)

{

prtHeaviside[j] = 0.5 * (1 + k3 * atan(prtPhi[j] / m_epsilon));

}

}

}

void duLevelSet::Curvature()

{

Mat dx, dy;

Sobel(m_mPhi, dx, m_mPhi.depth(), 1, 0, 1);

Sobel(m_mPhi, dy, m_mPhi.depth(), 0, 1, 1);

for (int i = 0; i < m_iRow; i++)

{

float *prtdx = (float *)dx.ptr(i);

float *prtdy = (float *)dy.ptr(i);

for (int j = 0; j < m_iCol; j++)

{

float val = sqrtf(prtdx[j] * prtdx[j] + prtdy[j] * prtdy[j] + 1e-10);

prtdx[j] = prtdx[j] / val;

prtdy[j] = prtdy[j] / val;

}

}

Mat ddx, ddy;

Sobel(dx, ddy, m_mPhi.depth(), 0, 1, 1);

Sobel(dy, ddx, m_mPhi.depth(), 1, 0, 1);

m_mCurv = ddx + ddy;

}

void duLevelSet::BinaryFit()

{

Heaviside();

float sumFG = 0;

float sumBK = 0;

float sumH = 0;

Mat temp = m_mHeaviside;

Mat temp2 = m_mImage;

float fHeaviside;

float fFHeaviside;

float fImgValue;

for (int i = 1; i < m_iRow; i++)

{

float *prtHeaviside = &(m_mHeaviside.at<float>(i, 0));

uchar *prtImgValue = &(m_mImage.at<uchar>(i, 0));

for (int j = 1; j < m_iCol; j++)

{

fImgValue = prtImgValue[j];

fHeaviside = prtHeaviside[j];

fFHeaviside = 1 - fHeaviside;

sumFG += fImgValue*fHeaviside;

sumBK += fImgValue*fFHeaviside;

sumH += fHeaviside;

}

}

m_FGValue = sumFG / (sumH + 1e-10);

m_BKValue = sumBK / (m_iRow*m_iCol - sumH + 1e-10);

}

int duLevelSet::EVolution(std::vector<Mat>& vecSIIter)

{

float fCurv;

float fDirac;

float fPenalize;

float fImgValue;

double dLastArea = 0;

int iter_times = 0;

for (int it = 0; it < m_iterNum; it++)

{

Dirac();

Curvature();

BinaryFit();

filter2D(m_mPhi, m_mPenalize, m_depth, m_mK, Point(1, 1)); // △φ

for (int i = 0; i < m_iRow; i++)

{

float *prtCurv = &(m_mCurv.at<float>(i, 0));

float *prtDirac = &(m_mDirac.at<float>(i, 0));

float *prtPenalize = &(m_mPenalize.at<float>(i, 0));

uchar *prtImgValue = &(m_mImage.at<uchar>(i, 0));

for (int j = 0; j < m_iCol; j++)

{

fCurv = prtCurv[j];

fDirac = prtDirac[j];

fPenalize = prtPenalize[j];

fImgValue = prtImgValue[j];

float lengthTerm = m_nu* fDirac * fCurv; // Length constraints

float penalizeTerm = m_mu*(fPenalize - fCurv); // Penalty

float areaTerm = fDirac * m_lambda1 * // Global term

(-((fImgValue - m_FGValue)*(fImgValue - m_FGValue))

+ ((fImgValue - m_BKValue)*(fImgValue - m_BKValue)));

m_mPhi.at<float>(i, j) = m_mPhi.at<float>(i, j) + m_timestep*(lengthTerm + penalizeTerm + areaTerm);

}

}

cvtColor(m_mImage, showIMG, CV_GRAY2BGR);

Mat Mask = m_mPhi >= 0;

dilate(Mask, Mask, Mat(), Point(-1, -1), 3);

erode(Mask, Mask, Mat(), Point(-1, -1), 3);

findContours(Mask,

cons_out,// output

RETR_EXTERNAL,

CHAIN_APPROX_NONE);

drawContours(showIMG, cons_out, -1, Scalar(255, 0, 0), 1);

Mat matIter = Mat::zeros(showIMG.size(), showIMG.type());

showIMG.copyTo(matIter);

vecSIIter.push_back(matIter);

double dNowArea = duContours2::GetTotalArea(cons_out);

double dAreadiff = abs(dNowArea - dLastArea);

{

if (dAreadiff < dLastArea * 0.01 || dNowArea <= 1)

{

if (iter_times <= 0)

iter_times = it;

}

}

dLastArea = dNowArea;

}

return iter_times;

}

}
